# Open Science for Veterinary Education Research

**DOI:** 10.3389/fvets.2021.745779

**Published:** 2021-09-09

**Authors:** Jill R. D. MacKay

**Affiliations:** Veterinary Medical Education Division, Royal (Dick) School of Veterinary Studies, University of Edinburgh, Edinburgh, United Kingdom

**Keywords:** education, interdisciplinary research, education research, open science, methodology

## Introduction

The “reproducibility crisis” (also known as the replication crisis) is the name given to a phenomenon of concern in research. It describes the recent findings that many pieces of published research may not be reliable, or even claimed as “false findings” (1). There may be many underlying reasons for this, from the innocent: a lack of understanding of statistical analysis or research design (2, 3); to the concerning: researchers may be consciously or unconsciously obscuring or concealing findings to publish research that may otherwise not be of publishable quality (4–6). In practice, this has resulted in many eye-catching claims published in high-impact journals being shown to be poorly replicated (7). The reproducibility crisis first gathered steam in the psychological and applied behavioral sciences (8). Itis now receiving attention in the “harder” sciences, including quantum computing (9) and there has been large-scale investigation of replication in various cancer biology findings (10).

**Figure 1:**
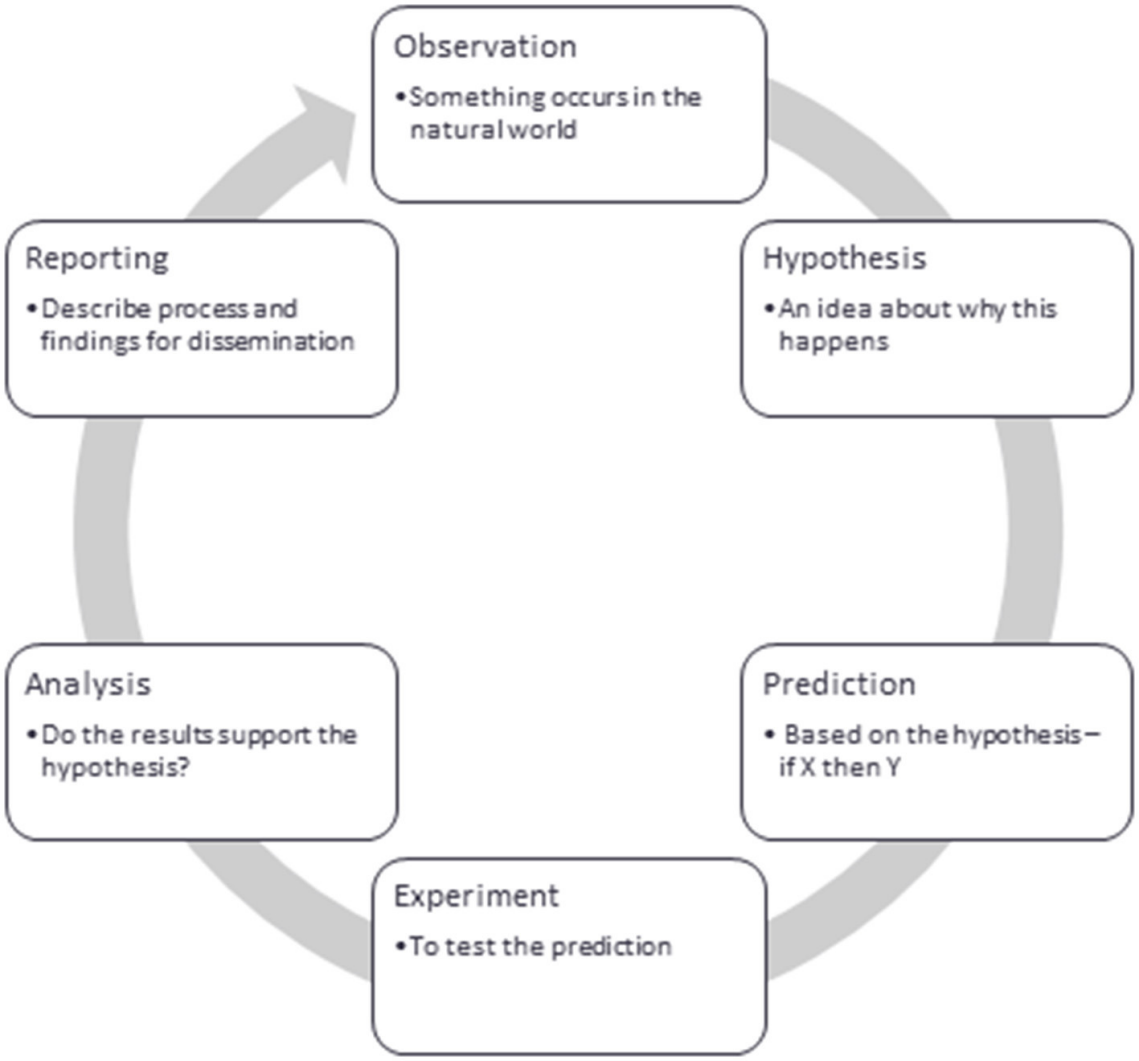
A generalized outline of the hypothetico-deductive model for scientific discovery.

Many researchers, particularly those educated in highly positivistic or “hard” science models (11) are familiar with the “hypothetico-deductive” model (Figure 1) of the scientific method (12).

This model, alongside the highly incentivised drive for publication [“publish or perish” (13)], is vulnerable to the HARKing practice [hypothesizing after results are known (14)], which has been speculated to be a fundamental cause of the reproducibility crisis (15). After running a study and exploring the results, researchers may be tempted to retrospectively create a hypothesis to fit their findings.

## Does VetEd Need Open Science?

Veterinary education research is a necessarily interdisciplinary area of research, requiring an understanding of veterinary science, educational research, psychology, and often many other aspects, such as business research or animal welfare and ethology.

Education research, which is strongly related to the terms Scholarship of Teaching and Learning (SoTL) and Discipline Based Educational Research (DBER), explores the practices around learning and teaching to improve the experience and outcomes for educators and students (16, 17). In practice, these fields all draw from educational psychology and reflective and philosophical practice to create a truly interdisciplinary field of study (18). In veterinary education research, we are mainly interested in whether veterinary students are becoming competent veterinarians, and their learning experience in that journey. The potential for doubting veterinary education research findings is one that requires careful scrutiny.

In general, veterinary education research is aiming to create as many excellent vets as possible with a positive learning experience. This is not always easy. What is “excellent” and how do we define “most”? How do we measure attainment or enjoyment? Is the students' experience in the classroom more important than what their degree enables them to do after? Issues of validity and reliability in veterinary education are already intensively discussed in veterinary assessment (19, 20), but to the author's knowledge, there have been no published reports on the reproducibility of research findings in veterinary education. We do know, however, that within the veterinary education community, there are concerns regarding the quality of research (21), particularly in how best to support new researchers into the discipline. It may be fair to say that this is a good time in the field of veterinary research to reflect on our practice and explore what can be done to facilitate better research in future. With more veterinary programmes opening in the United Kingdom (22), we are well-placed to pay attention to the developments in related fields, and make use of their own experiences to improve the quality and experience of the veterinarians under our care.

Questionable research practices have been defined (5) as including: failing to report all of a study's dependent measures, failing to report all of a study's conditions, collecting more or less data during analysis to support a hypothesis, rounding *p*-values, claiming to have expected unusual findings (HARKing), claiming that demographics have no impact on data, and outright falsification of data. These practices have recently been found to be prevalent among education researchers (23). It is important here to ensure that the push to open research practices does not turn into a witch hunt. While there is little peer-reviewed literature on this, there are some concerning reports that the open research movement may negatively impact researchers careers (24). These concerns should be heard, and act as a guide for how we respond to unsettling claims about questionable research practices. These findings should be viewed as feedback with which we can make better decisions as a field. Open science practices are a “carrot” which should incentivise us to produce more replicable, more robust findings, not a “stick” that will punish researchers for falling out of line.

## What Can VetEd Do With Open Science?

The manifesto for reproducible science (15) details a range of approaches that can be used to support more open research practices. For veterinary education, there are a number that can be integrated into our current practice.

### Improve Methodology

The manifesto promotes improving methodological training and facilitating access to methodology support. Due to the interdisciplinary nature of veterinary education research it is important that this includes collaborations with relevant practitioners. Collaboration is another key element of the manifesto. This should be supported by vet schools, and supported through the community of practice built up around veterinary education research [see (21, 25)]. Supporting research across fields and schools can support multi-site comparisons and replication across different cohorts. In light of its interdisciplinary nature, it is particularly important for veterinary education research to consider methodology. Many researchers may not be familiar with the various assumptions and biases inherent within theoretical stances, and feel uncomfortable evaluating or conducting research outside of their own wheelhouse. The flow of ontology to epistemology to theoretical perspective to methodology and methods (26) is often not made clear in researcher training, or indeed in the reporting studies, which is often subject to undisclosed and unrecognized bias (27, 28). Good quality qualitative research is not necessarily replicable in the standard framing of the replication crisis [although a characterization of replication is a study which could be considered diagnostic evidence for an existing claim (29)]. Instead, good qualitative research often aims to produce useful models for future work (30, 31). Veterinary education research should be aware of the strengths and limitations of different methodological approaches, and recognize no one approach can capture the entirety of the human experience. While individual researchers cannot be expected to master the gamut of research approaches that are applicable to veterinary education research, the field should aim to cultivate a range of approaches, seeking out collaboration where appropriate, and peer review from experts in methodology alongside subject expertise.

### Reporting and Dissemination

To facilitate the clearer reporting of findings, pre-registering, sharing analyses, and pre-printing can be useful tools. Pre-registering is the practice of declaring the research design and intended analyses prior to collecting data. It is very similar to what is requested in most ethical applications, and aims to prevent HARKing or other post data-collection tampering. Pre-registering does not preclude adapting studies, as you can log why and when changes to protocol were made. While pre-registering was initially considered for study designs more like randomized controlled trials, there are now a wide range of pre-registration templates including qualitative pre-registrations and open-ended registrations for large multi-design projects. A range of potential pre-registration templates can be found on the Open Science Foundation registries website: https://help.osf.io/hc/en-us/articles/360019738794-Select-a-Registration-Template.

Pre-registering can also encourage the sharing of analyses. This can be as simple as a description of what variables and what tests you intend to use. However, as more open-source powerful statistical softwares become available such as R, Python and Julia, it is becoming more possible to share full analyses. This allows other researchers to investigate every step of the process, and can also be a great advantage when the data itself cannot be shared, such as in the case of sensitive student data (32, 33). The production and sharing of reproducible workflows not only benefits the individual researcher, but supports the reproducibility of findings overall (34).

To further support dissemination, researchers can also make use of meta-data such as the OrcID (Open Research & Contributor ID) which allows for tracking of research across changes of name, institution, and different archival systems, eliminating ambiguity in citations (35). While not necessary, they are particularly valuable for early career researchers, or those who may be moving between disciplines, allowing them to “tag” their work across a range of specialities and institutions.

Data sharing is another aspect of reporting which supports openness within education research. While data sharing is highly prevalent in some fields, there are complex ethical considerations regarding human data within social science contexts (32, 36). Where participants are informed and have consented to share their data, and where reasonable precautions are taken regarding ethical concerns (37), sharing data can help reduce unnecessary data collection, support the development of researchers in areas like the Global South (38), and help to catch errors within the research process (39).

Finally, dissemination and reporting can be further improved through pre-printing, the process of making articles available prior to peer-review. Pre-printing has a host of benefits (40, 41) including enhancing sight of the findings and facilitating open review, improving the transparency of peer review, and facilitating the publication of controversial findings. Pre-printing also allows for the sharing of author's final version manuscripts, as they can be updated post peer-review. This will support the availability of research beyond paywalls. Unfortunately, not all journals support pre-printing. In the author's experience, both Medical Teacher and Journal of Veterinary Medical Education have in 2020–2021 discouraged the use of pre-printing by considering it prior-publication, thus making pre-printed papers unable to be published by those journals. However, other journals, such as Frontiers in Veterinary Science support the use of open publishing approaches. Researchers must be cautious in pre-printing to ensure they are not inadvertently cutting themselves off from their desired audience, but should also participate in journal communities to encourage pre-printing where appropriate.

## Discussion and Conclusions

Veterinary education research is a field which is continuing to grow and develop. It is well-placed to learn from the reproducibility crises in psychology and medicine, and to integrate these lessons into its own practice. Reproducibility is an essential component of good, robust research. This is particularly important in a field like veterinary education research which seeks to produce competent veterinarians. It is vital we continue to look to other fields and incorporate their hard-won revolutions into our own practice.

## Author Contributions

The author confirms being the sole contributor of this work and has approved it for publication.

## Conflict of Interest

The author declares that the research was conducted in the absence of any commercial or financial relationships that could be construed as a potential conflict of interest. The author is a moderator for EdArXiv, which is an unpaid voluntary role.

## Publisher's Note

All claims expressed in this article are solely those of the authors and do not necessarily represent those of their affiliated organizations, or those of the publisher, the editors and the reviewers. Any product that may be evaluated in this article, or claim that may be made by its manufacturer, is not guaranteed or endorsed by the publisher.

## References

[1] Ioannidis JPA. Why most published research findings are false. PLoS Med. (2005) 2:e124. 10.1371/journal.pmed.0020124 16060722 PMC1182327

[2] Fanelli D. Is science really facing a reproducibility crisis, and do we need it to? Proc Natl Acad Sci USA. (2018) 115:2628–31. 10.1073/pnas.1708272114 29531051 PMC5856498

[3] Gilbert DT King G Pettigrew S Wilson TD. Comment on “Estimating the reproducibility of psychological science.” Science. (2016) 351:1037. 10.1126/science.aad7243 26941311

[4] Spellman BA. Introduction to the special section: data, data, everywhere … especially in my file drawer. Perspect Psychol Sci. (2012) 7:58–9. 10.1177/1745691611432124 26168423

[5] John LK Loewenstein G Prelec D. Measuring the prevalence of questionable research practices with incentives for truth telling. Psychol Sci. (2012) 23:524–32. 10.1177/0956797611430953 22508865

[6] Simmons JP Nelson LD Simonsohn U. False-positive psychology: undisclosed flexibility in data collection and analysis allows presenting anything as significant. Psychol Sci. (2011) 22:1359–66. 10.1177/0956797611417632 22006061

[7] Camerer CF Dreber A Holzmeister F Ho T-H Huber J Johannesson M. Evaluating the replicability of social science experiments in Nature and Science between 2010 and 2015. Nat Hum Behav. (2018) 2:637–44. 10.1038/s41562-018-0399-z 31346273

[8] Open Science Collaboration. PSYCHOLOGY. Estimating the reproducibility of psychological science. Science. (2015) 349:aac4716. 10.1126/science.aac4716 26315443

[9] Frolov S. Quantum computing's reproducibility crisis: Majorana fermions. Nature. (2021) 592:350–2. 10.1038/d41586-021-00954-8 33846620

[10] Errington TM Iorns E Gunn W Tan FE Lomax J Nosek BA. An open investigation of the reproducibility of cancer biology research. Elife. (2014) 3:1–9. 10.7554/eLife.04333.004 25490932 PMC4270077

[11] Staller KM. Epistemological boot camp: the politics of science and what every qualitative researcher needs to know to survive in the academy. Qual Soc Work. (2012) 12:395–413. 10.1177/1473325012450483

[12] Davidoff F. We need better ways to create new hypotheses and select those to test. BMJ. (2012) 345:1–2. 10.1136/bmj.e7991 23187096

[13] Clapham P. Publish or perish? Bioscience. (2005) 55:390–1. 10.1641/0006-3568(2005)0550390:POP2.0.CO;2

[14] Kerr NL. HARKing: hypothesizing after the results are known. Perosnal Soc Psychol Rev. (1998) 2:196–217. 15647155 10.1207/s15327957pspr0203_4

[15] Munafò MR Nosek BA Bishop DVM Button KS Chambers CD Percie N. A manifesto for reproducible science. Nat Publ Gr. (2017) 1:1–9. 10.1038/s41562-016-0021 33954258 PMC7610724

[16] O'Brien M. Navigating the SoTL landscape: a compass, map and some tools for getting started. Int J Scholarsh Teach Learn. (2008) 2:1–20. 10.20429/ijsotl.2008.020215

[17] MacKay JRD. Discipline based education research for animal welfare science. Front Vet Sci. (2020) 7:7. 10.3389/fvets.2020.00007 32047758 PMC6997439

[18] Miller-Young J Yeo M. Conceptualizing and communicating SoTL : a framework for the field. Teach Learn Inq. (2015) 3:37–53. 10.20343/teachlearninqu.3.2.37

[19] Hecker K Violato C. Validity, reliability, and defensibility of assessments in veterinary education. J Vet Med Educ. (2009) 36:271–5. 10.3138/jvme.36.3.271 19861713

[20] Rodríguez-Martínez H. Quality commitment and management in veterinary education. J Vet Med Educ. (2006) 33:165–71. 10.3138/jvme.33.2.165 16849291

[21] Baillie S Rhind S MacKay J Murray L Mossop L. The VetEd conference: evolution of an educational community of practice. J Vet Med Educ. (2021) e20200154. 10.3138/jvme-2020-0154. [Epub ahead of print]. 34097582

[22] Anon. Two universities join up to announce plans for new West Midlands vet school. Vet Rec. (2017) 181:78. 10.1136/vr.j3503 28733492

[23] Makel MC Hodges J Cook BG Plucker JA. Both questionable and open research practices are prevalent in education research. Educ Res. (2021). 10.3102/0013189X211001356

[24] Pownall M Talbot CV Henschel A Lautarescu A Lloyd K Hartmann H. Navigating open science as early career feminist researchers. PsyArXiv [Preprint]. (2020). 10.31234/osf.io/f9m47

[25] Baillie S Rhind S Mossop L MacKay J. VetEd: The Evolution of an Educational Community of Practice. (2019). p. S2–P11. Available online at: https://app.oxfordabstracts.com/events/785/program-app/submission/115378 (accessed Dec 10, 2019).

[26] Crotty M. Introduction: the research process. In: The Foundations of Social Research Meaning and Perspective in the Research Process. London: SAGE Publications (1998). p. 1–17.

[27] Braun V Clarke V. Using thematic analysis in psychology. Qual Res Psychol. (2006) 3:77–101. 10.1191/1478088706qp063oa

[28] Twining P Heller RS Nussbaum M Tsai CC. Some guidance on conducting and reporting qualitative studies. Comput Educ. (2017) 106:A1–9. 10.1016/j.compedu.2016.12.00234429309

[29] Nosek BA Errington TM. What is replication? PLoS Biol. (2020) 18:e3000691. 10.1371/journal.pbio.3000691 32218571 PMC7100931

[30] Noble H Smith J. Issues of validity and reliability in qualitative research. Evid Based Nurs. (2015) 18:34–5. 10.1136/eb-2015-102054 25653237

[31] Smith B. Generalizability in qualitative research: misunderstandings, opportunities and recommendations for the sport and exercise sciences. Qual Res Sport Exerc Heal. (2018) 10:137–49. 10.1080/2159676X.2017.1393221

[32] Mackay JRD. Freeing the free-text comment : exploring ethical text mining in the higher education sector. In: Analysing Student Feedback in Higher: Using Text-Mining to Interpret the Student Voice Education. Taylor & Francis (2021). (In press).

[33] MacKay JRD. On the horizon: making the best use of free text data with shareable text mining analyses. J Perspect Appl Acad Pract. (2019) 7:57–64. Available online at: https://jpaap.napier.ac.uk/index.php/JPAAP/article/view/354

[34] Kitzes J Turek D Deniz F. The practice of reproducibility: case studies and lessons from the data-intensive sciences. The Practice of Reproducible Research, In: Kitzes J Turek D Deniz F editors. Oakland, CA: University of California Press (2018).

[35] Haak LL Fenner M Paglione L Pentz E Ratner H. ORCID: a system to uniquely identify researchers. Learn Publ. (2012) 25:259–64. 10.1087/20120404

[36] Ross MW Iguchi MY Panicker S. Ethical aspects of data sharing and research participant protections. Am Psychol. (2018) 73:138–45. 10.1037/amp0000240 29481107

[37] Bishop L. Ethical sharing and reuse of qualitative data. Aust J Soc Issues. (2009) 44:255–72. 10.1002/j.1839-4655.2009.tb00145.x

[38] Serwadda D Ndebele P Grabowski MK Bajunirwe F Wanyenze RK. Open data sharing and the Global South—Who benefits? Science. (2018) 359:642–3. 10.1126/science.aap8395 29439233

[39] Bishop D. Data Sharing may Lead to Some Embarrassment but will Ultimately Improve Scientific Transparency and Accuracy. | Impact of Social Sciences [Internet]. LSE Blog. 2014 [cited 2020 Dec 18]. Available online at: https://blogs.lse.ac.uk/impactofsocialsciences/2014/05/29/data-sharing-exciting-but-scary/

[40] Bourne PE Polka JK Vale RD Kiley R. Ten simple rules to consider regarding preprint submission. PLoS Comput Biol. (2017) 13:e1005473. 10.1371/journal.pcbi.1005473 28472041 PMC5417409

[41] Ginsparg P. Lessong from arXic's 30 years of information sharing. Nat Rev Phys. (2021) 1–2. 10.1038/s42254-021-00360-z 34377944 PMC8335983

